# Exposure to Chemotherapy During Childhood or Adulthood and Consequences on Spermatogenesis and Male Fertility

**DOI:** 10.3390/ijms21041454

**Published:** 2020-02-20

**Authors:** Marion Delessard, Justine Saulnier, Aurélie Rives, Ludovic Dumont, Christine Rondanino, Nathalie Rives

**Affiliations:** EA 4308 “Gametogenesis and Gamete Quality”, Normandie Univ, UNIROUEN, Rouen University Hospital, Biology of Reproduction–CECOS Laboratory, 76000 Rouen, France; marion.delessard@etu.univ-rouen.fr (M.D.); justine.saulnier@etu.univ-rouen.fr (J.S.); aurelie.rives@chu-rouen.fr (A.R.); ludovic.dumont1@univ-rouen.fr (L.D.); christine.rondanino@univ-rouen.fr (C.R.)

**Keywords:** chemotherapy, fertility, adulthood exposure, childhood exposure

## Abstract

Over the last decade, the number of cancer survivors has increased thanks to progress in diagnosis and treatment. Cancer treatments are often accompanied by adverse side effects depending on the age of the patient, the type of cancer, the treatment regimen, and the doses. The testicular tissue is very sensitive to chemotherapy and radiotherapy. This review will summarize the epidemiological and experimental data concerning the consequences of exposure to chemotherapy during the prepubertal period or adulthood on spermatogenic progression, sperm production, sperm nuclear quality, and the health of the offspring. Studies concerning the gonadotoxicity of anticancer drugs in adult survivors of childhood cancer are still limited compared with those concerning the effects of chemotherapy exposure during adulthood. In humans, it is difficult to evaluate exactly the toxicity of chemotherapeutic agents because cancer treatments often combine chemotherapy and radiotherapy. Thus, it is important to undertake experimental studies in animal models in order to define the mechanism involved in the drug gonadotoxicity and to assess the effects of their administration alone or in combination on immature and mature testis. These data will help to better inform cancer patients after recovery about the risks of chemotherapy for their future fertility and to propose fertility preservation options.

## 1. Introduction

In recent years, the number of cancer survivors has continuously increased thanks to earlier detection of cancer and progress in cancer treatment and medical care. In the United States, nearly 14.5 million cancer survivors have been identified in January 2014 and this number will probably reach 19 million by January 2024 [1]. Using data from 153 cancer registries in 62 countries, a study reports that the incidence of cancer in children under the age of 14 was 140 per million persons per year during 2001–2010 [2]. In France, approximately 2200 new cases of cancer are diagnosed each year among children and adolescents [3,4]. These cancers are the second most frequent cause of death for children under 15 years of age. Leukaemia (29%), mainly acute lymphoblastic leukaemia, is among the most common childhood cancer in addition to central nervous system tumours (23%) and lymphoma (12%) [4]. The five-year survival rate of children and adolescents with cancer has improved significantly in recent decades, with nearly 82% for all types of cancer [3]. Among adolescents and young adults, 2400 cases of cancer have been registered during 2000–2008. In this population, the most frequently observed cancers are lymphomas (23%) including Hodgkin’s lymphomas (17,8%) and non-Hodgkins lymphoma (4.4%), germ cell tumours (16.7%), melanomas (10.9%), and thyroid cancers (10.9%) [3].

The improvement of the quality of life of cancer survivors has thus become a major public healthcare issue. Cancer treatment-related toxicities can generate late and long-term side effects in cured patients and infertility could be one of them [5,6]. In fact, the prevalence of male infertility is 46% in adult survivors of childhood cancer versus 17.5% in siblings [7]. Nevertheless, given the urgency of diagnosis and the implementation of effective therapy for children, the toxicity of cancer treatments on the gonad was overshadowed for a long time. Moreover, earlier studies suggested that the childhood period provide protection against chemotherapy-induced gonad damage owing to the supposed “quiescent stage” of the testis during this period. Recent studies have shown that the testis can be affected by cancer treatments at all stages of life, and consequently of development [8,9]. However, very few studies concerning the gonadotoxicity of anticancer drugs in adult survivors of childhood cancer are available compared with those concerning the effects of chemotherapy exposure during adulthood.

In order to increase the effectiveness of cancer treatments, combinations of molecules are frequently used such as MOPP (mechloretamine, vincristine, procarbazine, and prednisone) and ABVD (adriamycin, bleomycin, vinblastine, and dacarbazine) for the treatment of Hodgkin’s lymphoma and CHOP (cyclophosphamide, doxorubicin, vincristine, and prednisolone) for non-Hodgkins lymphoma. In most cases, the regimen of treatment received by children, adolescents, and young adults contains alkylating agents (e.g., chlorambucil, cyclophosphamide, cisplatin, busulphan), which are associated with a high risk of infertility [10,11]. Most chemotherapy molecules have been classified according to their gonadotoxic risk, that is, according to the degree of spermatogenesis impairment ranging from oligozoospermia to non-obstructive azoospermia after recovery (Table 1) [12,13]. Thus, DNA intercalating agents (e.g., irinotecan, daunorubicin, doxorubicin, etoposide, bleomycin) that induce or stabilize DNA strand breaks, antimetabolites (e.g., methotrexate, cytarabine) that impact DNA biosynthesis, and spindle poisons (e.g., vincristine, vinblastine) that interact with tubulin have been considered as moderate and low risk for fertility [14,15]. This classification indicates the risks of infertility after chemotherapy exposure during childhood or adulthood and helps clinicians to better refer patients for fertility preservation option. However, this classification remains questionable as it appears to be difficult to identify the risk of each molecule, generally administered in combination. Moreover, it should be noticed that very few data are available on the gonadotoxicity of chemotherapy administered during the prepubertal period and most of them are deduced from adult studies [14].

In the current review, the epidemiological and experimental data about the effects of chemotherapy exposure during the prepubertal period or adulthood on spermatogenesis, sperm production, sperm nuclear quality, and offspring’s health are presented.

## 2. Consequences of Chemotherapy Exposure During Childhood on Future Male Fertility

The testes have a dual function: an exocrine function via the production of spermatozoa in the seminiferous tubules and an endocrine function via the secretion of testosterone by Leydig cells in the interstitial compartment. Thus, the testis is traditionally divided into two interdependent compartments: the seminiferous tubules—the site of spermatogenesis—and the interstitial tissue—containing Leydig cells and blood capillaries [16]. Spermatogenesis is a process of cell division and differentiation leading to the production of spermatozoa from puberty to senescence. It takes place inside the seminiferous tubules in the testis, which are composed of Sertoli and germ cells. This process can be divided into three phases after puberty: (i) spermatogonial proliferation; (ii) spermatocyte meiosis leading to the production of haploid spermatids; and (iii) spermiogenesis, during which spermatids differentiate into spermatozoa. The duration of spermatogenesis differs according to species: 74 days are required to obtain spermatozoa in humans, 52 days in rats, and 35 days in mice [17].

Spermatogonial stem cells (SSCs) allow the continuous production of spermatozoa and support spermatogenesis. In the seminiferous tubules, Sertoli cells are known to maintain close interactions with SSCs and to form a proper microenvironment—the so-called “niche”—essential for their self-renewal and differentiation. In humans, undifferentiated spermatogonia are composed by A_dark_ and A_pale_ spermatogonia [18]. Spermatogonia A_dark_ form the quiescent reserve of the stem cell population, while maintaining their mitotic activity in the case of spermatogonia depletion. The A_pale_ population is mitotically active and able to differentiate into type B spermatogonia. Differentiating spermatogonia (type B) divide, through mitosis, gives spermatocytes [18]. In rodents, undifferentiated spermatogonia consist of A_single_ (SSCs), A_paired_, and A_aligned_. A_aligned_ spermatogonia differentiate into A1 spermatogonia that, in turn, give six generation of differentiating spermatogonia (A_1–4_, A_in_, B) [17,19]. Rats and mice are the most commonly used animal models to study the gonadotoxicity of chemotherapeutic molecules [14].

Childhood has been often considered as a quiescent period of testicular development, protecting the testes from the adverse effects of chemotherapy. Thus, very few data are available on the impact of chemotherapy exposure during childhood on testicular functions and most of them are deduced from adult studies. However, prepubertal human testis is quietly active with an increase in testicular volume, proliferation of immature Sertoli cells, and rise in the number of Leydig cells [20]. Successful establishment of these physiological modifications influences the pubertal development of the testis, and consequently adult fertility. Therefore, chemotherapy may disrupt them. It should be noted that most of the studies listed in the current review define “chemotherapy exposure during childhood”, including not only the administration of cancer treatment in prepubertal boys, but also in postpubertal adolescents.

### 2.1. Effects of Chemotherapy on Somatic Cells and Spermatogenic Progression in Adulthood

#### 2.1.1. Effects of Chemotherapy on Somatic Cells

Infertility observed after chemotherapy exposure can be the result of direct injury on germ cells or indirect damages on endocrine and paracrine control of somatic cells [21]. In fact, during infancy, Sertoli cells proliferate actively, which makes them a potential target of childhood cancer treatment toxicity [20]. Few data are available on the long-term impact of chemotherapy exposure during childhood on Sertoli cells. A case report has demonstrated the presence of immature Sertoli cells (expressed Cytokeratin-18 normally absent after puberty) in an azoospermic man who underwent chemotherapy treatment at 13 years old [22]. Moreover, reduced sperm counts found in male survivors of paediatric cancer were systemically associated with increased follicle stimulating hormone (FSH) levels, indirectly reflecting Sertoli cell alteration [8,23]. However, in contrast to germ cells, Sertoli cells seemed to develop a better resistance to chemotherapeutic agents in an animal model. In vitro studies using prepubertal rat testis showed no impact on the Sertoli cell number after 48 h exposure to doxorubicin, cisplatin, or cyclophosphamide [24]. Another study highlighted cell death resistance in primary Sertoli cells isolated from prepubertal rat, after 24 h in vitro exposure to cisplatin and etoposide. The Sertoli cell survival can be explained by a mechanism of apoptosis and autophagy inhibition [25]. However, an increased oxidative stress has been measured in an immature Sertoli cell line (Ser-W3) after doxorubicin exposure [26]. Moreover, the administration of DNA topoisomerase II inhibitor led to morphological and nuclear alterations, resulting in cytoplasmic vacuolization and abnormal chromatin condensation, respectively [27,28]. Sertoli cell dysfunction was observed after in vivo exposure to chemotherapeutic molecules with a decreased production of androgen binding protein (ABP) and transferrin, two proteins involved in the regulation of spermatogenesis [29,30].

Despite their important role in the spermatogenesis support and the maintenance of spermatogonial stem cell niche, little attention has been paid to myoid cells and Leydig cells and no studies have focused on macrophages [14]. To the best of our knowledge, only one experimental study investigated the peritubular myoid cells and demonstrated that the in vitro exposure to doxorubicin did not affect the proliferation of myoid cells in the rat testicular tissue [31].

Leydig cell function is impaired in survivors of paediatric cancer [23,32,33]. Some patients present a small drop of testosterone plasmatic level and an important increase of luteinizing hormone (LH) plasmatic level owing to an amplified response of LH to gonadotropin releasing hormone to compensate for Leydig cell dysfunction [33,34,35]. Many studies have reported the negative impact of chemotherapy on the gonad endocrine function. Indeed, childhood cancer survivors have an increased risk of hypogonadism compared with the overall population [36]. The estimation of the hypogonadism risk would improve patient care and ensure a better prevention of the long-term complications of androgen deficiency [36]. Although most children treated for cancer develop secondary sexual characteristics and enter into puberty at a normal age, some patients might display sex hormone deficiency, delayed onset of puberty, and small testicular volume, suggesting a potential spermatogenesis impairment in addition to testicular endocrine dysfunction [34,37,38]. In animal models, few data are available on the impact of chemotherapy exposure on Leydig cells. No change in Leydig cells function and morphology has been observed in the pre-pubertal testis after 48 h in vitro exposure to doxorubicin [31]. Similarly, recent data have reported that Leydig cell density was unaffected after 24 h in vitro exposure to cisplatin, cyclophosphamide, and doxorubicin [24]. Although no change has been observed in rodent Leydig cells in in vitro studies, elevated LH levels found in survivors of paediatric cancer might suggest Leydig cell alteration; the few studies available do not currently allow conclusions to be drawn about the impact of chemotherapy exposure during childhood on testicular somatic cells.

#### 2.1.2. Effects of Chemotherapy on Spermatogonia and Spermatocytes

Exposure to alkylating agents during childhood has been particularly associated with testicular damages characterized by Sertoli cell only tubules, reduced tubular diameter, and interstitial fibrosis [39,40]. Moreover, a long-term reduction of the SSC pool has been observed in prepubertal patients exposed to cancer treatment including alkylating agents, and the risk of infertility appears to increase with the proportion of altered SSCs [11,41]. Most of the time, the SSC pool is not totally depleted and the surviving germ cells allow spermatogenesis recovery [42,43]. Similarly, in adult rats treated with low daily doses of etoposide during the prepubertal period (30- to 60-day-old rats), testicular weight is reduced and testicular tissues display severe alterations with Sertoli cell only tubules persisting in 113-day-old rats [44]. It should be noted that most of the data concerning prepubertal chemotherapy exposure are based on studies with 25–30-day-old rats or 14-day-old mice corresponding to an exposure during the first wave of spermatogenesis. Very few data are available on the impact of the administration of chemotherapy before entry into meiosis (before day 7–8 in mouse and around day 12 in rat model) [45,46] (Figure 2). Therefore, a depletion of spermatogonia characterized by Sertoli cell only tubules has been observed in testicular tissue from five-day-old mouse cultured in vitro after exposure to the irinotecan metabolite SN38 [47]. With the same culture condition, a significant loss of SSCs after cyclophosphamide, vincristine, and doxorubicin exposure at concentrations used in humans has been reported [24]. Two in vivo studies have shown that tyrosine kinase inhibitors such as imatinib mesylate, when administered during the early postnatal period in rodent, impair the formation of the SSC pool and reduce the proliferation of type A spermatogonia [48,49]. Indeed, this treatment used as a first-line treatment of chronic myeloid leukaemia and gastrointestinal stromal tumors inhibits platelet-derived growth factor receptor β subtype activity, which plays a major role in the proliferation and migration of gonocytes, a significant step to maintain their survival and to generate the SSC pool [48,50,51]. Imatinib affects neither SSC self-renewal nor their capacity to colonize seminiferous tubules and initiate spermatogenesis, which might explain the normal sperm count found in the epididymis of exposed animals after an 11-week recovery period [49,52]. In addition, the Comet assay highlights an increase in DNA breaks in a cell line with SSC characteristics following exposure to doxorubicin, considered to have a moderate risk on fertility [53]. Likewise, a short-term exposure to chemotherapeutic drugs such as bleomycin, etoposide, doxorubicin, cisplatin, and cyclophosphamide results in DNA damages (increased in γH2AX expression) in a C18–4 mouse spermatogonial cell line and in culture of mouse prepubertal testicular tissue after 48 h and 16 h, respectively [24,54]. In fact, telomere DNA damage, associated with an inhibition of telomerase activity, has been evidenced in C18–4 cell line exposed to cisplatin or cyclophosphamide [54]. Because these structures are critical for genetic stability, their dysfunction might affect the most differentiated germ cell types and contribute to infertility [55]. Moreover, the accumulation of DNA damages, following in vivo administration of a low dose of etoposide in prepubertal rats, might lead to the activation of the apoptotic pathway and the reduction in the number of primary differentiated spermatogonia and spermatocytes [56]. In addition, it has been hypothesized that unrepaired DNA damage in SSCs, occurring after exposure to chemotherapy during the prepubertal period (i.e., 30-day-old in rats), might be maintained in spermatogonia throughout spermatogenesis cycles, and consequently might impact sperm quality [57].

### 2.2. Effects of Chemotherapy on Spermatozoa and Offspring

#### 2.2.1. Sperm Production and Recovery Period

Paediatric cancer treatments commonly contain alkylating agents. The use of the cyclophosphamide equivalent dose (CED) is recommended to quantify the exposure to alkylating agents and a negative correlation has been reported between the CED and sperm concentration in a cohort of adult male survivors of childhood cancer [10,58]. A study, based on a large cohort of 214 patients treated for cancer between 1970 and 2002, reports that 25% of adults who received alkylating agents during childhood display an azoospermia. Moreover, normozoospermic patients (48%) display impairment of sperm motility and morphology [10]. Another study including patients treated during the same period as the previous one (1970–2002) has shown that the percentage of azoospermic patients reached 50% in men diagnosed for Hodgkin’s lymphoma before 10 years of age, 17% for non-Hodgkin’s lymphoma, and 12% for leukemia. Moreover, 67% of patients treated with a sterilizing dose of alkylating agents (i.e., doses higher than values given in Table 1) display azoospermia, but none are observed in cancer survivors receiving lower doses of alkylating agents [59]. Impairment of fertility depends not only on the type of cancer and disease stage, but mostly on the therapy regimen.

#### 2.2.2. Sperm Nuclear Abnormalities

Very few studies focus on sperm nuclear abnormalities. Three studies have explored sperm DNA integrity in childhood cancer survivors. In adult survivors of childhood cancer, sperm DNA and chromatin integrity seem to be unaffected by chemotherapy treatment [60,61]. However, chemotherapy exposure during adolescence seems to promote epimutations and to alter DNA methylation in spermatozoa [62].

DNA damages have been highlighted in animal models. Head abnormalities in almost 50% of spermatozoa, associated with disruption of chromatin compaction, have been observed in adult mice who received cyclophosphamide treatment at 14 days old [63]. Moreover, DNA strand breaks are also found in spermatozoa following doxorubicin treatment of 30-day-old rats [57]. Data on nuclear sperm quality, after exposure to chemotherapy during childhood, remain limited in humans as well as in animal models.

#### 2.2.3. Effects of Chemotherapy on Cancer Survivors’ Offspring

Male survivors who received high doses of alkylating drugs during childhood are especially less likely to father a child [64,65]. Offspring of survivors diagnosed in childhood do not appear to have a higher risk of congenital abnomalities, genetic diseases, and abnormal karyotypes compared with their siblings [66,67,68,69]. Moreover, no significant difference has been observed in the risk of hospitalization between children of paediatric cancer survivors and the overall population [69].

In animal models, there is also a notable lack of data on intergenerational and transgenerational effects of paternal exposure during childhood on the progeny [70]. Etoposide administration during the prepubertal period (25-day-old rats) reduces the number of offspring obtained from the mating of treated adult rats with fertile females [56]. Moreover, the in vitro treatment of spermatogonial cells (C18–4 mouse spermatogonial cells) with cisplatin and preactivated analog of cyclophosphamide lead to a reduction of telomere length and inhibition of telomerase activity [54]. Disruption of germ cell telomeres leads to aberrant fertilization and abnormal cleavage of embryos, which might cause an increase of embryo losses or developmental abnormalities [71]. In this context, it would be necessary to examine the health and reproductive functions of the descendants of paediatric cancer survivors.

## 3. Consequences of Chemotherapy Exposure During Adulthood on Male Fertility

### 3.1. Effects of Chemotherapy on Somatic Cells and Spermatogenesis Recovery

#### 3.1.1. Effects of Chemotherapy on Somatic Cells

The successful completion of spermatogenesis depends, among others, on a normal endocrine balance. In men with cancer history, assessment of testicular function is achieved by measuring hormone levels of FSH, LH, and testosterone in blood sample [72,73]. These biomarkers have been measured to estimate testicular damage due to cancer treatment particularly in the case where semen analysis was not possible. In clinical practice, the measurement of serum testosterone and LH has been used to evaluate Leydig cell impairment [74]. It is more difficult to assess Sertoli cell function owing to the lack of reliable measurable biomarkers. Most of the time, Sertoli cell dysfunction is deducted from a rise in the FSH level associated with spermatogenic failure [75]. Some studies suggest to assess the inhibin/FSH ratio because Sertoli cells are the main source of inhibin in the testis and it could be a circulating marker for Sertoli cell function [76,77]. Following chemotherapy, adult men with haematological malignancies show a decrease of inhibin B associated with a rise in the FSH level, suggesting Sertoli cell damage and the loss of negative feedback control of FSH [77]. However, it appears to be difficult to evaluate Sertoli cell damage using only the inhibin/FSH index, because of the close interactions between the gonadotropic axis and Sertoli and Leydig cells. Therefore, it would be better to assess the pattern of serum inhibin/FSH, in combination with other hormonal factors such as testosterone and LH [76].

The administration of cyclophosphamide to adult mice leads to a disturbance of protein expression in Sertoli cells [78]. This alkylating agent notably causes a reduction in the production of glial cell-derived neurotrophic factor, involved in SSCs self-renewal and spermatogonia differentiation. Moreover, the expression of occludin, a main component of tight junctions essential for the blood–testis barrier functionality, is diminished, whereas the expression of transforming growth factor β3, a down-regulator of tight junctions, is increased [78]. The disruption of protein expression of Sertoli cells as a result of chemotherapy exposure might impair spermatogenesis.

Leydig cells, which secrete testosterone, have often been considered more resistant to chemotherapy than germ cells and Sertoli cells. However, it has been shown that Leydig cell function can be impaired in long-term cancer survivors associated with a raise of LH blood level and a low or normal testosterone concentration [32,79]. Moreover, it has been hypothesized that the alteration of the seminiferous epithelium might lead to a decrease of testicular volume and blood flow and, consequently, limited the testosterone level leaving the testis [80]. This reduction of blood flow may be compensated by Leydig cells by increasing intratesticular testosterone concentration. In addition, reduced arterial flow leads to a disruption of paracrine control of Leydig cells and to a reduction of the stimulatory response to LH [81]. In the adult rat testis, it has been observed that bleomycin, etoposide, and cisplatin (BEP) chemotherapy induces an oxidative stress status, Leydig cell hyperplasia, and inhibition of the transcription of genes encoding steroidogenic enzymes. A decline in *CYP19A1* gene expression (enzyme involved in the conversion of testosterone into estrone and estradiol) associated with an increase in the Leydig cell population could explain the low/normal testosterone levels [82].

As in prepubertal testis, data on the impact of chemotherapy on somatic cell are mainly limited to Sertoli and Leydig cells. No study investigated the impact of anticancer treatment on macrophages, despite their role as “guardians of fertility” [83]. To the best of our knowledge, only Sasso-Cerri et al. have shown a nuclear fragmentation and a depletion of peritubular myoid cells in adult rat testes after alkylating agent exposure [84].

#### 3.1.2. Effects of Chemotherapy on Spermatogonia and Spermatocytes

The toxicity of chemotherapy on spermatogonial and spermatocytes is difficult to investigate in adult male without testicular biopsy and few data are available on biomarkers of spermatogonial or spermatocyte damage. Anti-Müllerian hormone (AMH) has been proposed as a marker for chemotherapy-induced testicular toxicity in combination with FSH, testosterone, and inhibin B [85]. Thus, an increase of serum AMH has been observed in patients (average age was 38 years) six months after treatment with alkylating agents, while this hormone generally decreased with the onset of testicular maturation. In adults, inhibin B would consist of α-subunit produced by Sertoli cells and β_B_-subunit provided by spermatocytes and spermatids, and decreases AMH production by inhibiting the effect of FSH on Sertoli cells [86,87]. The reduction of spermatocytes and spermatids caused by chemotherapy decreases the production of inhibin B. The negative feedback control of FSH is disrupted and consequently causes an increase in the level of AMH [85]. Studies on larger cohorts are necessary to define the role of AMH and its use as a biomarker of testicular toxicity, as it has been proposed for assessing ovarian toxicity after chemotherapy exposure [88].

In cancer survivors, poor sperm quality (reduced sperm count and motility, increased abnormal forms) can persist several years after the completion of the cancer treatment, suggesting that the effects of cancer drugs on SSCs have a long-term impact [89]. It is reasonable to assume that SSC injuries could persist throughout spermatogenic cycles. Because of their scarcity and their difficulty to be purified, very few studies have investigated SSC damages after chemotherapy exposure. Cancer treatment leads to a reduction in the number and proliferative activity of rat SSCs [90]. Moreover, in a rat spermatogonial cell line with SSC characteristics (GC-6spg), an increased expression of CDKN1a, a protein involved in cell cycle arrest and gene expression involved in DNA repair has been evidenced after doxorubicin exposure [53,91]. The cytotoxic effects of chemotherapeutic compounds have been also evaluated in undifferentiated spermatogonia in rats. Thus, undifferentiated A_aligned_ spermatogonia are severely depleted after in vivo BEP administration compared with undifferentiated A_single_ and A_paired_ [90].

Spermatocytes are also sensitive to the cytogenotoxic effect (i.e., oxidative stress, DNA damage, apoptosis) of chemotherapeutic compounds. The exposure to cancer drugs during adulthood leads to an increase in the number of seminiferous tubules devoid of germ cells associated with a reduction of seminiferous tubule diameter, and epithelial vacuolization involves a decreased testis weight in rodents [92,93]. Numerous chemotherapeutic compounds induce an increased oxidative stress in testicular tissues associated with a downregulation of anti-oxidant enzymes, which are necessary to prevent the excess formation of reactive oxygen species (ROS) [94,95,96]. This rise of oxidative stress might result in DNA damage in germ cells and accumulation of DNA injuries leads to germ cell death. Cyclophosphamide induces, for instance, apoptosis in spermatogonia and spermatocytes [97]. Germ cell apoptosis might be responsible for oligozoospermia or azoospermia, but also prevents the production of spermatozoa with DNA damages and their potential transmission to the offspring [97]. However, it has been shown in animals treated by chemotherapy that damaged germ cells could escape from apoptosis and lead to abnormal sperm production [98]. It has been hypothesized that the increased expression of the protooncogene *Jun* and a disruption in cell cycle checkpoints could explain the survival of abnormal germ cells after the administration of the BEP cocktail [98]. Besides altering gene expression, some chemotherapeutic agents could also interfere with the DNA replication process or chromosome segregation. This is the case of etoposide, a topoisomerase II inhibitor, which prevents the religation of DNA double strands after replication, leading to chromosomal fragmentation, and inhibits the normal segregation of homologous chromosomes, resulting in aneuploidy in mouse post-meiotic cells [99].

### 3.2. Effects of Chemotherapy on Spermatozoa and Offspring

#### 3.2.1. Sperm Production and Recovery Period

In a study assessing the semen quality of cancer patients before and after chemotherapy, a decrease in sperm concentration, motility, and semen volume and an increase in sperm abnormal forms have been described [100]. The patients treated for leukaemia, lymphoma, or a testicular cancer presented different degrees of azoospermia or oligozoospermia, and approximately 63% of them developed irreversible azoospermia [100]. Despite major impairments of fertility after treatment, several studies have reported a recovery period that might vary between individuals and depending on the stage of disease development and the therapy regimen [101]. In fact, one year after treatment, patients who have been treated by more than two BEP cycles displayed a lower total sperm count, whereas men treated by one or two BEP cycles had a normal sperm recovery (≤39 × 10^6^/ejaculate) after testicular cancer treatment [102]. The recovery period also depended on the dose received by the patient, mainly for doses of alkylating agent. For instance, a return to normozoospermic levels was observed in 70% of men who received cumulative doses of cyclophosphamide under 7.5 g/m^2^, whereas only 10% recovered with higher doses after five years or even more [103]. It has been shown that 100% of the patients of reproductive age treated for Hodgkin’s lymphoma with MOPP chemotherapy were azoospermic 14 months after the treatment [104,105]. The earliest sperm recovery has occurred 15 months after completion of the treatment, whereas some patients still presented an azoospermia 20 years after. Although the patients recover spermatogenesis, most of them display low sperm counts [105]. Another treatment, the ABVD regimen, which is less gonadotoxic, is proposed to treat Hodgkin’s lymphoma. No change has been observed in sperm concentration after one year in 90% of men treated with ABVD [106]. These studies highlight that it would be necessary to wait for several cycles of spermatogenesis before recovery of pre-treatment sperm counts, at least one year or more than four cycles of spermatogenesis after the end of the cancer treatment.

In addition to disrupting sperm production, chemotherapeutic agents might also affect semen quality. An in vitro study performed on freshly ejaculated human sperm has shown that doxorubicin and vincristine, regarded as molecules with intermediate and low risk of gonadotoxicity, respectively, had a sperm-immobilizing activity [107]. An increase in fructose and citric acid concentrations in the seminal fluid has also been reported during and after the treatment with chlorambucil for chronic lymphocytic leukaemia, Hodgkin’s, and non-Hodgkin’s lymphoma. One year after treatment, the patients recovered a normal sperm count, as well as fructose and citric acid concentrations within normal range [108].

Experimental data have also reported a decrease in semen quality (sperm concentration and motility) in adult rats exposed to adriamycin, CHOP, and BEP treatment [109,110,111]. In rats, the administration of CHOP leads to a reduction in sperm count and sperm recovery has been observed nine weeks after treatment or the equivalent of more than one spermatogenesis cycle [112]. However, the recovery of a normal sperm count does not guarantee an intact sperm nuclear quality [113].

#### 3.2.2. Sperm Nuclear Abnormalities

While spermatogenesis might recover after completion of chemotherapy, persistent nuclear damages are found in spermatozoa of a majority of cancer survivors [89,114,115]. Indeed, a significant level of sperm DNA and chromatin damages has been observed in the semen samples of cancer patients, even after a 24-month recovery period [89,116]. Nuclear abnormalities observed in ejaculated sperm appeared to vary according to the stage of differentiation (spermatogonia, spermatocytes, or spermatids) at the time of chemotherapy exposure and the anticancer agent used [117].

Cancer treatments induced aberrant chromosome segregation, leading to the production of sperm with numerical chromosome abnormalities. In patients treated for testicular cancer with BEP regimen, total sperm aneuploidy rate, evaluated using fluorescence in situ hybridization (FISH) for chromosomes 8, 12, 18, X, and Y, significantly increased six months after the treatment [118]. Likewise, another study including five patients treated by BEP has shown an increase of the frequencies of disomy and diploidy for chromosome 16 and 18 during a recovery period from 6 to 18 months [119]. In addition, a raised frequency of chromosome 13 and 21 nullisomy has been observed in testicular cancer patients and Hodgkin’s lymphoma patients 18 to 24 months after the chemotherapy initiation [120]. Most of studies using FISH analysis report a sexual chromosome disomy in patients after CHOP/MOPP-ABV, ABVD, or BEP regimen [115,120,121]. Close to 40% of testicular cancer patients did not recover normal sperm aneuploidy rates after treatment, and it would be advisable to postpone the parental project up to 24 months after more than two BEP chemotherapy cycles to prevent a potential risk of aneuploid conceptus [115]. Chemotherapeutic treatments have deleterious impacts on sperm chromosome constitution depending on the therapy received and the delay after the end of treatment. Thus, a multicenter prospective study assessing sperm aneuploidy in lymphoma patients demonstrated that ABVD and CHOP/MOPP-ABV (mechlorethamine, vincristine, procarbazine, prednisone-doxorubicin, bleomycin, vinblastine) treatments resulted in increased aneuploidy frequencies three months after the completion of the treatment. In patients treated with ABVD, aneuploidy rates returned to lower values than before treatment one or two years following the chemotherapy, whereas these rates were still relatively high until two years after in patients who received CHOP/MOPP-ABV [121].

The epidemiological data on sperm aneuploidy match with the experimental results obtained in rodent models. Indeed, it has been reported that etoposide treatment in mice led to an increased frequency of sperm aneuploidy 49 days after the end of chemotherapy [99,122]. It would appear that exposure of pachytene spermatocytes to etoposide led to the increased frequencies of chromosome numerical and structural abnormalities (duplications and deletions) in mice spermatozoa, whereas SSCs exposure only lead to sperm chromosome structural abnormalities [122].

After oncological treatment, some studies show no change in sperm DNA fragmentation index in patients treated for testicular germ cell tumours, as well as Hodgkin’s and non-Hodgkin’s lymphomas [123]. However, a rise of sperm DNA fragmentation assessment by COMET and TUNEL assay is observed in testicular cancer and Hodgkin’s lymphoma individuals after a recovery period of more than 24 months [73,89]. DNA fragmentation might be responsible for the abnormal chromatin compaction detected with sperm chromatin structure assay in testicular cancer patients [116]. Chemotherapeutic molecules such as alkylating agents modified DNA base and inducted DNA cross-links that have been highlighted in many tissues in cancer patients [124,125]. In rat testis, DNA adducts have been observed after cisplatin exposure and could explain DNA strand breaks in spermatozoa [126]. In fact, a higher proportion of sperm with DNA single strand breaks and cross-links has been reported in adult rats exposed to a chronic alkylating agent treatment [111,127]. The presence of sperm DNA damages has been correlated with abnormal chromatin compaction [128,129]. The administration of BEP to rats induced defective sperm chromatin compaction, with the presence of low levels of protamine 1 and high levels of histones within the sperm nuclei [130]. BEP treatment disturbs histone H4 hyperacetylation, which was essential for the recruitment of a testis-specific bromodomain-protein BRDT necessary for histone removal and binding of transition proteins and protamines [131]. Moreover, the higher proportion of histone H3 monomethylated on lysine 9 (H3K9me) and the decreased proportion of the testis-specific histone H2B observed after BEP treatment might prevent chromatin decondensation in pachytene spermatocytes [132]. The high degree of chromatin packaging improves sperm motility and plays a key role in DNA protection because mature spermatozoa are deprived of repair mechanisms [133].

DNA methylation profiles has been impaired in 46% of oligozoospermic patients [134]. Hypomethylation of *H19*, a paternally imprinted gene, has been observed almost a year after the first day of initial treatment in one patient diagnosed for anaplastic oligodendroglioma [135]. Following the administration of cancer drugs, a reduced expression of DNA methyltransferases and disrupted DNA methylation patterns has been reported in rodent spermatozoa [136,137]. Hypomethylation on cytosine-guanine dinucleotide regions is the most frequent change observed after doxorubicin treatment. Therefore, the DNA methylation status of these regions could be used for the early diagnosis of testicular toxicity [137]. Several studies have shown that maintenance of DNA methylation is required to ensure the successful development of male germ cells and embryos in rodent models [138,139,140]. Despite erasure of epigenetic signatures during developmental reprogramming, some paternal epigenetic aberrations induced by anticancer treatments persist and could affect the offspring’s development [141].

#### 3.2.3. Effects of Chemotherapy on Cancer Survivors’ Offspring

The epidemiological data concerning the impact of paternal exposure to chemotherapy on the progeny are limited. A recent study performed on a large number of treated patients has shown a decreased likelihood of becoming a father, which depended on the location of the tumour, age, and the delay since diagnosis, compared with the background population [65]. In this study, it appears that chemotherapeutic treatments influence the birth rates (i.e., number of live child births), but not the mortality rates (i.e., offspring death after birth) [65]. The transmission of genetic damages to the children of cancer survivors was mainly studied by investigating congenital anomalies. It seemed that no increased risk of congenital anomalies has been observed among children parented by male cancer survivors in comparison with the offspring born from parents with no history of cancer [142]. Moreover, whole genome sequencing of germline, used to identify de novo alterations, reported no increase in de novo genetic events in children born after or before paternal exposure to chemotherapy [143]. However, this study is limited to two families, and thus needs to be confirmed on a larger cohort of patients.

Numerous data concerning the effects of paternal exposure to chemotherapy on the progeny are available in rodent models [70]. The experimental data report that anticancer drugs used separately or in combination result in increased preimplantation and postimplantation losses [110,111,112,144]. Disruption of zygotic gene activation has been found in embryo sired by a father treated by cyclophosphamide. This disruption of zygotic gene expression disturbs the kinetics of embryonic development and could explain the losses of early embryos fathered by males treated by alkylating agents [145]. In addition to embryo losses, studies performed in rats have shown a rise in postnatal mortality in litters descended from cyclophosphamide-treated fathers and grandfathers [146,147]. Moreover, one- to two-week cisplatin treatment was sufficient to induce growth retardations in the offspring sired by treated males. Although the risk of congenital anomalies was low, external malformations, such as omphalocele and micrognathia, were observed seven to nine weeks after paternal exposure [147]. In addition, cisplatin has long-term adverse effects on rat male and female reproductive development. The female offspring displayed a decrease in the number of germ cells in foetal ovaries, a decline of serum concentration of FSH, and dysregulation in the estrous cyclicity at adulthood. However, puberty onset, sexual behaviour, and fertility were not affected by paternal exposure to cisplatin [148]. Moreover, paternal exposure to cisplatin during peri-puberty influenced the timing of testicular descent, epididymal sperm counts, and testicular histology in the rat male offspring. It is important to point out that testicular histological alterations (Sertoli cell vacuolization, germ cell loss and multinucleate giant cell formation) observed in the adult offspring sired by treated males were similar to those seen in male rats treated directly with cisplatin [149].

Very few studies have investigated the F2 offspring sired by a grandfather treated by anticancer drugs. As observed for F1 offspring, pioneering studies have demonstrated that the increases in postimplantation losses, postnatal mortality rates, and number of malformed foetuses were also observed in the second generation [146,150]. A diminished learning capacity and spontaneous activity were evidenced in the F2 generation [151]. Because the males and females fathered by the alkylating agent-treated male were mated together in these studies, it cannot be concluded whether the mother, the father, or both transmitted chemotherapy effects. A more recent study demonstrated that anticancer drugs, other than alkylating agents, have affected reproductive parameters such as testis weight and seminiferous epithelium (vorinostat) in F2 and sperm vitality in F3 (vorinostat and decitabine), but did not affect the fertility of the F2 and F3 progeny. Sperm DNA methylation patterns of a few genes were affected in the F2 and F3 offspring after father’s treatment [152].

Although few studies are available, experimental data have reported a possible transgenerational transmission of the effects of paternal chemotherapy exposure on offspring, which needs to be investigated.

## 4. Conclusions

**Figure 1:**
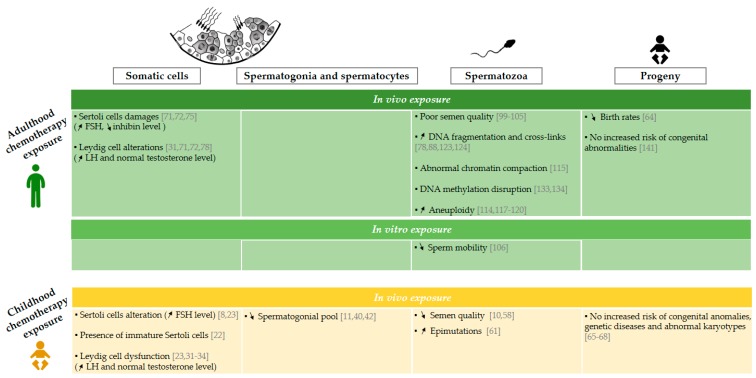
Effects of chemotherapy exposure during adulthood and childhood on men fertility. Schematic representation of epidemiological data available on the impact of the administration of chemotherapy on somatic cells [8,22,23,31,32,33,34,71,72,75,78], spermatogonia and spermatocytes [10,40,42], spermatozoa [10,58,61,78,88,99,100,101,102,103,104,105,106,114,115,117,118,119,120,123,124,133,134], and progeny [64,65,66,67,68,141] following chemotherapy administration during childhood and adulthood. “Childhood chemotherapy exposure” is defined as the administration of cancer treatment during infancy and adolescence period too. The arrows ↗ and ↘ indicate an increase and a decrease respectively. FSH: follicle stimulating hormone, LH: luteinizing hormone, SSC: spermatogonial stem cell.

**Figure 2:**
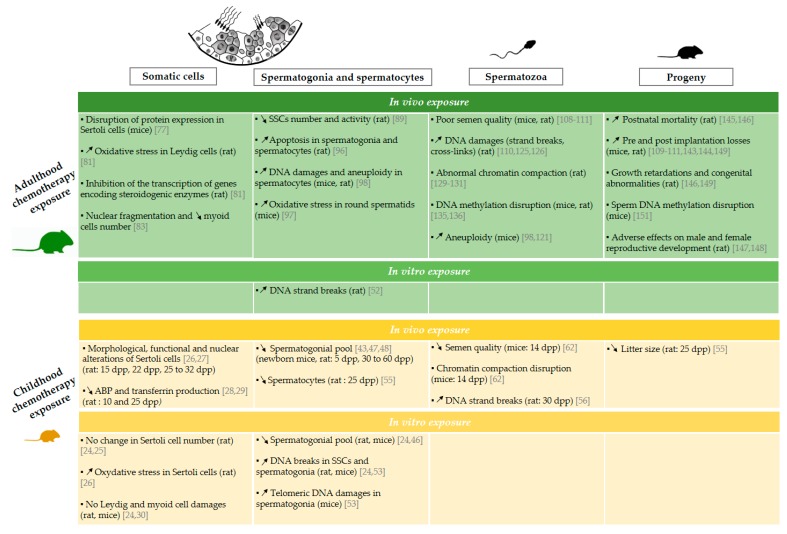
Effects of chemotherapy exposure during adulthood and childhood on male fertility in an animal model. Schematic representation of experimental data available on the impact of the administration of chemotherapy on somatic cells [24,25,26,27,28,29,30,77,81,83], spermatogonia and spermatocytes [24,43,46,47,48,52,53,55,89,96,97,98], spermatozoa [56,62,98,108,109,110,111,125,126,129,130,131,135,136] and progeny [55,109,110,111,143,144,145,146,147,148,149,151] following chemotherapy administration during childhood and adulthood. Animal species and the age at which the chemotherapy treatments achieved are specified in parentheses. The arrows ↗ and ↘ indicate an increase and a decrease respectively. ABP: androgen binding protein, SSC: spermatogonial stem cell, d*pp*: day post-partum.

Infertility is one of the potential adverse side effects of cancer treatments. Epidemiological and experimental studies are numerous and clearly describe the risk of infertility after anticancer treatment, especially in men who have been diagnosed in adulthood. Recent studies have raised awareness of the sensitivity of the immature testicular tissue to chemotherapy exposure and an increased risk of infertility in adult men treated during childhood compared with the overall population. This review highlighted the limited data available on the impact of chemotherapy exposure before puberty, especially with regard to the impact on somatic cells, interstitial tissue, and sperm quality in both human (Figure 1 [8,10,11,22,23,31,32,33,34,40,42,58,61,64,65,66,67,68,71,72,75,78,88,99,100,101,102,103,104,105,106,114,115,117,118,119,120,123,124,133,134,141]) and animal models (Figure 2 [24,25,26,27,28,29,30,43,46,47,48,52,53,55,56,62,77,81,83,89,96,97,98,108,109,110,111,121,125,126,129,130,131,135,136,143,144,145,146,147,148,149,151]). 

Most of the studies defined “childhood chemotherapy exposure” as the administration of cancer treatment during infancy and adolescence, and only four studies investigated the consequences of exposure before the first wave of spermatogenesis in rodents. The classification of chemotherapy molecules according to their low, moderate, or high risk on fertility remained questionable because it appeared difficult to identify the risk of each molecule, as they are often administered in combination. Moreover, most of these data are deduced from adult studies and may not be applicable to prepubertal testis. In this way, it is necessary to conduct further in vivo and in vitro studies on humans and animal models. Studies on animal models are a good way to investigate the mechanism of action of chemotherapy molecules as well as they are administered alone or in combination. In rodents, the most frequently studied treatments are MOPP for treatment of Hodgkin’s disease, CHOP for non-Hodgkin’s lymphoma, and BEP for testicular cancer, but to the best of our knowledge, no studies have examined combined treatment used for leukemias yet—the most common childhood cancer.

Although cancer treatments are improving to reduce side effects, fertility preservation procedures are still needed in males of all ages [153]. For postpubertal males producing sperm, sperm cryopreservation represents an easy and efficient fertility preservation method. However, for prepubertal boys, sperm collection is impossible and fertility preservation can be considered using testicular tissue freezing. Several experimental methods could be envisaged to use frozen-thawed immature testicular tissue in order to produce spermatozoa that can be used in assisted reproductive techniques: (i) in vitro maturation of SSCs (in vitro spermatogenesis); (ii) in vivo maturation of SSCs in the patient after autologous SSC transplantation or testicular tissue grafting; and possibly (iii) in vivo maturation of SSCs in animals after xeno-transplantation or testicular tissue grafting [154,155]. Although some methods, such as testicular tissue grafting, are encouraging, they will remain limited to certain types of cancer patients, with a low risk of locating tumour cells at the testicular level. Thus, in vitro spermatogenesis appears to be the most appropriate approach to avoid the potential risk of reintroducing tumour cells in the cured patient. The three-dimensional cell culture and the organotypic culture have been shown to support the differentiation of spermatogonia into spermatozoa in the rat and mouse model [156,157,158,159]. Moreover, the generation of sperm-like cells has been reported in 3D cell culture from spermatogonial cells of busulfan-treated mice [160]. Today, only the organotypic culture allows to produce mature spermatozoa and generate a healthy offspring by intracytoplasmic sperm injection (ICSI) in mice [158,161]. In humans, studies are still limited and demonstrated a maturation arrest at the post-meiotic stage after 3D culture and organotypic culture from prepubertal and pubertal testicular tissue of cancer patient who has or has not received gonadotoxic treatment [162,163,164,165]. These data may pave the way for future studies aimed to optimise the conditions for in vitro maturation of frozen-thawed testicular tissue for future use in fertility preservation strategies. Alternatively, less invasive methods are also in the experimental phase of development such as the administration of cytoprotective agent concomitantly to cancer treatment regimen or the miRNA replacement approaches to prevent chemotherapeutic damages [57,92,166,167,168,169].

Therefore, further research on the impact of chemotherapy on long-term spermatogenesis progression and maintenance, gamete quality, fertility, and progeny is urgently required. These data will help to better inform the paediatric cancer patients about the risks of chemotherapy for their future fertility and to propose fertility preservation options.

## Figures and Tables

**Table 1:** Risks of the main anticancer drugs on men fertility diagnosed in adulthood or in childhood.

Agents (Cumulative Dose for Effect)	Class of Anticancer Drugs	Risk on Fertility after Adulthood Exposure [12]	Risk on Fertility after Childhood Exposure [13]
Chlorambucil (1.4 g/m^2^)	Alkylating agent		High
Cyclophosphamide (19 g/m^2^)		High	High (7.5 g/m^2^)
Procarbazine (4 g/m^2^)		(Prolonged azoospermia)	High
Melphalan (140 mg/m^2^)			High
Cisplatin (500 mg/m^2^)			High
Busulfan (600 mg/kg)	Alkylating agent		High
Ifosfamide (42 g/m^2^)		Moderate	High (4 g/m^2^)
Carmustin (300 mg/m^2^)		(Likelihood of azoospermia, but always given with other sterilizing agents)	Low
Dactinomycin	DNA Intercalating		Low
Carboplatin (2 g/m^2^)	Alkylating agent		Moderate
Thiotepa (400 mg/m^2^)			Moderate
Doxorubicin (770 mg/m^2^)	DNA Intercalating		Moderate
Cytarabine (1 g/m^2^)	Antimetabolite		Moderate
Vinblastine (50 g/m^2^)	Spindle poison		Low
Vincristine (8 g/m^2^)			Low
		Low	
Dacarbazine	Alkylating agent	(Temporary reduction in sperm counts)	Moderate
Daunorubicin	DNA Intercalating		Moderate
Mitoxantrone			Moderate
Bleomycin	DNA strand breaks inducer		Low
Etoposide	Topoisomerase II inhibitor		Low
Fludarabine	Antimetabolite		Unknown
Fluorouracil			Low
Mercaptopurine			Low
Methotrexate			Low
Thioguanine			Unknown

## Abbreviations

MOPP	Mechloretamine, Vincristine, Procarbazine, and Prednisone
ABVD	Adriamycin, Bleomycin, Vinblastine, and Dacarbazine
CHOP	Cyclophosphamide, Doxorubicin, Vincristine, and Prednisolone
DNA	DeoxyriboNucleic Acid
SSCs	Spermatogonial Stem Cells
FSH	Follicle Stimulating Hormone
ABP	Androgen Binding Protein
LH	Luteinizing Hormone
γH2AX	γ Histone 2AX
dpp	day post-partum
CED	Cyclophosphamide Equivalent Dose
BEP	Bleomycin, Etoposide, and cisPlatin
CYP19A1	Cytochrome P450 Family 19 Subfamily A Member 1
AMH	Anti-Müllerian Hormone
CDKN1a	Cyclin-Dependent Kinase Inhibitor 1a
ROS	Reactive Oxygen Species
FISH	Fluorescence In Situ Hybridization
ABV	Adriamycin, Bleomycin, and Vinblastine
MOPP-ABV	Mechlorethamine, Vincristine, Procarbazine, Prednisone-Doxorubicin, Bleomycin, Vinblastine
TUNEL	TdT-Mediated dUTP Nickend Labelling Assay
BRDT	Testis-Specific Bromodomain-Protein
H3K9me	Histone H3 Monomethylated On Lysine 9
H2B	Histone 2B
H3	Histone 3
H4	Histone 4
H19D	Histone 19
ICSI	Intracytoplasmic Sperm Injection
